# Household availability of dietary fats and cardiovascular disease and mortality: prospective evidence from Russia

**DOI:** 10.1093/eurpub/ckab128

**Published:** 2021-07-30

**Authors:** Denes Stefler, Elvire Landstra, Martin Bobak

**Affiliations:** 1 Department of Epidemiology and Public Health, University College London, London, UK; 2 Department of Population Health Sciences, German Center for Neurodegenerative Diseases (DZNE), Bonn, Germany

## Abstract

**Background:**

The aim of this analysis was to examine the prospective association between household availability of lard, butter, margarine and vegetable oil with all-cause mortality and cardiovascular disease (CVD) incidence in a general population sample in Russia.

**Methods:**

Data from the Russian Longitudinal Monitoring Survey were used. 6618 adult individuals with no previous CVD who were recruited for the study in 1994 and followed-up in subsequent years were included in the analysis. Household availability of lard, butter, margarine and vegetable oil were assessed at baseline with questions on whether these food items were purchased by the participants’ family. Self-reported information on heart attack or stroke (CVD) and death reported by another household member were used as outcome.

**Results:**

Over the median follow-up of 11 years, 1787 participants died or reported incident CVD. In the multivariable adjusted survival models, household availability of lard was significantly associated with the combined outcome of CVD incidence and/or death (OR in the high vs. no availability categories: 1.31; 95% CI: 1.05–1.62). The associations with butter (1.06; 0.93–1.20), margarine (1.18; 0.94–1.47) and vegetable oil (0.92; 0.80–1.06) were not statistically significant. When self-reported CVD and mortality were examined separately, the association regarding lard was particularly strong for CVD (1.52; 1.11–2.09).

**Conclusion:**

Our results suggest that lard, a dietary fat of animal origin traditionally used in Eastern European cooking, is of a particular concern regarding CVD risk. Replacing it with plant-based oils in cooking practices is strongly recommended.

## Introduction

Eastern European countries have the highest dietary cholesterol intake globally, and they are also among the world leaders for saturated fat consumption.^1^ Traditionally meat-rich diet and the popularity of using animal fats for cooking in Eastern European cuisine^2–4^ are likely to be responsible for this observation, and animal fats may also contribute to the poor population health and high cardiovascular disease (CVD) mortality rates of the region.^5^ However, dietary habits are changing, and cooking with vegetable oils has become more common in recent decades.^6^ In fact, increased consumption of vegetable oils has been hypothesized as an important contributing factor for the reduced CVD mortality rates in Poland and the Czech Republic during the 1990s.^7^^,^^8^ Nonetheless, individual-level evidence for the association of animal fat or vegetable oil intakes with CVD risk in Eastern Europe is limited, and to date no large-scale epidemiological study investigated this link in Russia.

While globally the number of studies that examine the relationship between dietary fats and oils in relation to chronic disease outcomes is excessive, there are specific types of fats on which the available evidence is scarce. For example, lard, which is a common ingredient in Eastern European cooking, is considerably under-researched and the evidence on the association between its consumption and CVD or other diseases is still very limited.^4^

Household Budget Surveys (HBSs) are often used to assess food availability in various populations. Considering that dietary fats and oils are usually used for cooking rather than consumed on their own, it is suggested that HBSs may be more suitable to capture the presence of these foods in diet compared to nutritional assessment methods measuring actual intakes.^9–11^

The aim of this analysis was to investigate the association between household availability of dietary fats and oils of animal and vegetable origin (lard, butter, margarine and vegetable oil) and risk of CVD or death from any cause using data from the Russian Longitudinal Monitoring Survey (RLMS).

## Methods

### Study sample

The RLMS is a series of nationally representative annual surveys initiated in 1992, with longitudinal data collection beginning in 1994, to obtain information on the health, social and economic welfare of Russian citizens.^12–14^ The study used multistage sampling to select households, of which all members were included in the sample and followed-up in subsequent years (response rate in 1994: 87%). The study was conducted according to the guidelines laid down in the Declaration of Helsinki and all procedures involving research study participants were approved by the Institutional Review Board at the University of North Carolina at Chapel Hill. RLMS is a publicly available source of data.

From the 11 290 participants who were recruited to the study in 1994, in our analysis we excluded individuals under 18 years old (*n* = 2946), those with previously diagnosed CVD at baseline (*n* = 376), no follow-up data (*n* = 1194), or with missing data on any of the four dietary exposure variables or other covariates (*n* = 156). As a result of these omissions, 6618 individuals were included in the analytical sample.

### Data collection and follow-up

Availability of lard, butter, margarine and vegetable oil was assessed using household budget survey-style questions, asking all participants once a year whether their family had purchased these food items in the previous week and what was the amount they had bought. For the purpose of this analysis, data on these purchase habits were used from the baseline year (1994).

Presence of CVD was assessed with two self-reported questions, asking participants whether they had been diagnosed with heart attack or stroke. This information was collected every year between 1994 and 2016, except 1997 and 1999. In addition, we used information on deaths of study participants reported by other household members. Follow-up time for each participant was calculated as the time in years between 1994 and the year when CVD diagnosis or death was reported (participant reached the end-point) or the year of last contact (participant censored), whichever came first. The median (IQR) follow-up time in the analytical sample was 11 (4–21) years.

In addition to the main exposure and outcome variables, information on age, sex, household size (number of individuals in the same household), smoking (regular smoker; ex-smoker; never-smoker), alcohol intake (consumed alcohol several times a week; once a week; less than once a week; never) and highest level of education (primary or less; secondary; vocational; higher degree) were also included in the analysis as potential confounders. Data on these variables were used from the baseline year and included in the analysis as time-fixed covariates.

### Statistical analysis

Based on the per person household availability of lard, butter, margarine and vegetable oil, participants were categorized into three groups regarding all four items: (i) no availability—individuals who did not buy the specific item; (ii) low availability; (iii) high availability. The cut-off between low and high availability was determined based on the median value for each food item, which were 0.5 kg/person for lard, 0.33 kg/person for butter, 0.25 kg/person for margarine and 0.66 l/person for vegetable oil.

The association between the examined fats and oils and self-reported CVD was assessed with discrete-time survival analysis, using the time in years between 1994 and 2016 as the underlying time variable.^15^ In order to take into account clustering of participants within families, robust standard errors were applied, adjusted for the household identifier variable.

The analysis was carried out in three multivariable adjusted models. In model 1, the examined associations were adjusted for age and sex. In model 2, they were further adjusted for education, alcohol intake frequency and smoking habits, while in model 3, the availability of the four examined dietary fats and oils were also adjusted for each other.

In addition to the main analysis, we also carried out two further sensitivity analyses. Firstly, we examined the above associations after excluding participants who died or reported CVD events in the first two years of follow-up. Secondly, we explored these associations with adjustments for additional potential confounders, such as BMI, marital status, income, self-reported health and previously diagnosed diabetes. Data from all these variables were used from the baseline wave of the study.

All analyses were carried out using Stata 15 statistical software (StataCorp, TX, USA).

## Results

Table 1 shows the descriptive characteristics of the sample at baseline. 8.4% of participants indicated that their family purchased lard in the previous week, while this proportion was 38.3%, 12.9% and 26.4% for butter, margarine and vegetable oil, respectively. Individuals in the high availability categories were the oldest for all four food items. In terms of sex, education, smoking and alcohol drinking habits, although we found some significant differences between the different availability groups, clear trends across categories were not apparent. Regarding the correlations with each other, we found mostly positive associations between the four examined food items.

**Table 1:** Descriptive characteristics of the sample

	**Lard availability** ^a^				**Butter availability** ^a^				**Margarine availability** ^a^				**Vegetable oil availability** ^a^			
	No	Low	High	***P*-value** ^b^	No	Low	High	***P*-value** ^b^	No	Low	High	***P*-value** ^b^	No	Low	High	***P*-value** ^b^
*n*	6065	305	248		4080	1365	1173		5765	544	309		4873	883	862	
**Age, mean**	44.8	42.9	50.0	<0.001	45.0	41.5	48.7	<0.001	45.2	41.7	45.8	<0.001	44.4	43.5	49.5	<0.001
** (SD)**	(16.8)	(16.4)	(17.0)		(17.0)	(15.3)	(17.1)		(16.9)	(15.7)	(16.4)		(16.7)	(16.2)	(17.1)	
**Females, %**	57.8	57.1	54.8	0.642	57.5	55.3	60.8	0.020	57.8	56.3	56.6	0.730	57.9	56.4	57.5	0.717
**Higher education, %**	16.5	19.0	15.7	0.070	15.7	19.1	16.6	<0.001	16.3	19.5	16.5	0.006	16.4	17.6	16.6	<0.001
**Regular smoker, %**	30.5	34.4	29.8	0.194	31.0	35.2	24.4	<0.001	30.4	33.6	31.7	0.074	30.8	35.0	25.4	<0.001
**Alcohol intake several times a week, %**	9.3	7.5	10.5	0.008	9.0	9.7	9.7	0.001	9.2	9.9	10.0	0.086	9.2	9.9	9.4	0.029
**High lard availability, %**	NA	NA	NA	NA	3.3	2.8	6.6	<0.001	3.7	4.8	3.6	<0.001	3.1	3.6	7.7	<0.001
**High butter availability, %**	17.1	19.7	31.1	<0.001	NA	NA	NA	NA	16.4	24.5	30.1	<0.001	15.6	19.4	28.1	<0.001
**High margarine availability, %**	4.6	7.2	4.4	<0.001	5.6	5.1	7.9	<0.001	NA	NA	NA	NA	3.6	6.5	8.8	<0.001
**High vegetable oil availability, %**	12.5	13.1	26.6	<0.001	11.4	11.4	20.6	<0.001	12.4	13.4	24.6	<0.001	NA	NA	NA	NA

NA, not applicable.

a The cut-off between low and high availability was determined based on the median value for each food item. The median values were 0.5 kg/person for lard, 0.33 kg/person for butter, 0.25 kg/person for margarine and 0.66 l/person for vegetable oil.

b *P*-values were calculated with ANOVA for age and Chi-square tests for all other variables.

The results regarding the associations between the examined dietary fats and oils with newly developed CVD or death are presented in table 2. In the fully adjusted models (model 3), we found strong positive association between household availability of lard and combined CVD or death, with 31% (95%CI: 5–62%) higher risk among those who bought large amounts of this fat compared to those who bought none of it. Regarding butter, margarine and vegetable oil, we found no significant associations with this outcome. When we examined self-reported CVD and mortality separately, the association for lard was stronger for CVD (OR: 1.52; 95%CI: 1.11–2.09) and weaker for mortality (1.11; 0.85–1.44).

**Table 2:** Association between lard, butter, margarine and vegetable oil availability and self-reported CVD or death

Outcome	Food product	Availability	*n* outcome per 1000 person-years	Model 1		Model 2		Model 3	
				OR	(95%CI)	OR	(95%CI)	OR	(95%CI)
Combined CVD incidence and all-cause mortality	Lard	No	1617/72.2	1.00	(ref.)	1.00	(ref.)	1.00	(ref.)
		Low	79/3.5	1.10	(0.88–1.37)	1.15	(0.92–1.42)	1.10	(0.88–1.39)
		High	91/2.5	1.28	(1.03–1.60)	1.30	(1.05–1.61)	1.31	(1.05–1.62)
	Butter	No	1086/48.1	1.00	(ref.)	1.00	(ref.)	1.00	(ref.)
		Low	342/16.6	1.10	(0.97–1.25)	1.12	(0.99–1.27)	1.09	(0.96–1.24)
		High	359/13.4	1.04	(0.92–1.17)	1.07	(0.94–1.21)	1.06	(0.93–1.20)
	Margarine	No	1570/68.3	1.00	(ref.)	1.00	(ref.)	1.00	(ref.)
		Low	127/6.5	1.02	(0.85–1.22)	1.04	(0.87–1.24)	0.99	(0.82–1.19)
		High	90/3.4	1.15	(0.92–1.44)	1.18	(0.94–1.47)	1.18	(0.94–1.47)
	Vegetable oil	No	1269/57.9	1.00	(ref.)	1.00	(ref.)	1.00	(ref.)
		Low	249/10.1	1.16	(1.00–1.34)	1.16	(1.00–1.33)	1.12	(0.97–1.30)
		High	269/10.3	0.91	(0.80–1.05)	0.95	(0.83–1.09)	0.92	(0.80–1.06)
CVD incidence	Lard	No	744/72.2	1.00	(ref.)	1.00	(ref.)	1.00	(ref.)
		Low	36/3.5	1.09	(0.78–1.51)	1.10	(0.80–1.53)	1.10	(0.79–1.54)
		High	49/2.5	1.54	(1.12–2.12)	1.52	(1.11–2.09)	1.52	(1.11–2.09)
	Butter	No	498/48.2	1.00	(ref.)	1.00	(ref.)	1.00	(ref.)
		Low	148/16.6	1.04	(0.86–1.25)	1.04	(0.86–1.25)	1.04	(0.85–1.26)
		High	183/13.4	1.15	(0.96–1.38)	1.15	(0.96–1.37)	1.13	(0.95–1.36)
	Margarine	No	732/68.3	1.00	(ref.)	1.00	(ref.)	1.00	(ref.)
		Low	53/6.5	0.91	(0.69–1.20)	0.90	(0.68–1.19)	0.87	(0.66–1.16)
		High	44/3.4	1.20	(0.86–1.68)	1.21	(0.87–1.68)	1.20	(0.86–1.68)
	Vegetable oil	No	590/57.9	1.00	(ref.)	1.00	(ref.)	1.00	(ref.)
		Low	105/10.1	1.06	(0.85–1.32)	1.04	(0.83–1.30)	1.02	(0.81–1.28)
		High	134/10.3	0.99	(0.82–1.20)	1.00	(0.83–1.22)	0.95	(0.78–1.16)
All-cause mortality	Lard	No	1098/76.5	1.00	(ref.)	1.00	(ref.)	1.00	(ref.)
		Low	49/3.7	1.02	(0.80–1.31)	1.09	(0.84–1.40)	1.01	(0.77–1.31)
		High	54/2.8	1.05	(0.80–1.39)	1.09	(0.84–1.43)	1.11	(0.85–1.44)
	Butter	No	729/51.0	1.00	(ref.)	1.00	(ref.)	1.00	(ref.)
		Low	247/17.5	1.20	(1.04–1.38)	1.24	(1.07–1.43)	1.19	(1.02–1.38)
		High	225/14.5	0.94	(0.82–1.09)	1.01	(0.87–1.17)	0.99	(0.85–1.16)
	Margarine	No	1054/72.4	1.00	(ref.)	1.00	(ref.)	1.00	(ref.)
		Low	92/6.8	1.10	(0.89–1.36)	1.16	(0.94–1.44)	1.10	(0.88–1.37)
		High	55/3.7	1.02	(0.79–1.31)	1.08	(0.84–1.37)	1.07	(0.84–1.37)
	Vegetable oil	No	848/61.3	1.00	(ref.)	1.00	(ref.)	1.00	(ref.)
		Low	178/10.6	1.25	(1.06–1.48)	1.26	(1.06–1.49)	1.21	(1.01–1.44)
		High	175/11.0	0.88	(0.75–1.03)	0.94	(0.81–1.09)	0.93	(0.79–1.09)

Model 1: adjusted for age and sex; Model 2: in addition to model 1, further adjusted for education, smoking and alcohol intake frequency; Model 3: in addition to model 2, the four dietary fats/oils were further adjusted for each other.

While the prospective design of our analysis minimizes the possibility of reverse causation, it is not impossible that poorer health may lead to higher intake of butter and lard, which would, to some extent, explain the observed associations. In order to examine this possibility, we conducted a sensitivity analysis where individuals who died or reported CVD in the first two years of the follow-up were excluded from the analytical sample (Supplementary table S1). The results were generally very similar to our main findings.

## Discussion

Using longitudinal data from a nationally representative general population survey in Russia, we examined the prospective association between household availability of dietary fats and oils with self-reported CVD and all-cause mortality between 1994 and 2016. Our results indicated higher risk of CVD among those who had access to high amounts of lard in their household, but there was no clear association with the availability of butter, margarine or vegetable oil.

Lard is the traditional cooking fat still widely used in several Eastern European countries, and its association with higher risk of CVD and cancer mortality has been reported in a recently published analysis with general population data from the Czech Republic, Poland and Russia.^4^ To our knowledge, apart from the above study, no previous works examined the consumption of lard in relation to chronic disease outcomes. In terms of its effect on blood lipids, a network meta-analysis of randomized controlled trials found that lard, together with butter, were ranked the least favourable in terms of low-density lipoprotein (LDL) and total cholesterol reduction among 13 examined dietary fats and oils.^16^ The unfavourable effect of this food product on blood lipid profiles is also confirmed by animal experiments.^17^

The available evidence on the association between butter intake and CVD risk is not consistent. A systematic review published in 2016 found relatively small or neutral overall associations of butter with all-cause mortality, CVD, and diabetes,^18^ and epidemiological studies published since this review have provided contradictory findings.^19–21^

Considering its high saturated fat and cholesterol content, the impact of regular lard consumption on CVD risk can be potentially explained by the traditional diet-heart hypothesis, although the validity of this theory has been disputed.^22^ Nonetheless, our results support the view that regular animal fat consumption increases the risk of CVD, implying that replacing their consumption with vegetable oils could be beneficial for health.^23^ Although in the RLMS there is no information available on what specific type of vegetable oil was purchased by the participants, data from FAOSTAT suggests^6^ that the most popular vegetable oil in Russia during the observed time period was sunflower seed oil. As lard remains a popular ingredient in Russian and other Eastern European culinary practices, and is often used for cooking or consumed on its own (i.e. on bread), this food product could be an obvious target for public health nutritional campaigns in the region.

Our analysis has several limitations. First of all, availability of specific food items in the household does not necessarily imply actual consumption. In fact, previous analysis found generally weaker correlation between HBS data and individual-level intakes regarding animal fats and vegetable oils compared to other food groups.^9–11^ However, these foods are mostly used for cooking and less frequently consumed on their own, and this makes them prone to reporting bias, as their intake as cooking ingredient is often ignored. Therefore, it is possible that HBS data reflects a better estimation of their intake than the information collected with 24 h recall, 7 d records or food frequency questionnaires. Even if the actual consumed amount is inaccurate, the ability of the tool to differentiate between low and high consumers is likely to be valid, which is key to assessing their associations with health outcomes. The fact that our results are mostly in line with previous evidence from individual-level studies^4^^,^^18^ also supports the view that dietary data collected with HBS on animal fats and vegetable oils could be used as proxy for actual intake in epidemiological analyses.

Another limitation related to the food availability data stems from the fact that information on food purchases was collected for a one-week period which may not be representative to the participants’ regular purchase habits and therefore the general availability of the examined food items in the household. This issue is common to other dietary assessment tools that measure food intakes or availability at specific time points (i.e. 24 h recall)^24^, and needs to be considered in all nutritional epidemiological analyses where such methods are used.

It is likely that misclassification also applies to the measurement of the outcome. CVD incidence was assessed by self-report, and an objective confirmation of this information (i.e. doctor diagnosis) was not available. Similarly, some deaths may have been missed due to its reporting by other household members. Nonetheless, heart attack and stroke are major life events that are likely to be remembered if someone experiences them, and previous study has found high agreement between self-report and medical record for these conditions.^25^ Furthermore, the relative specificity of the associations for CVD (stronger effect for CVD incidence than for all-cause mortality of which about half of all deaths are non-CVD) further supports the overall plausibility of the findings.

While we adjusted our models for other lifestyle factors and education, the role of residual confounding in the observed associations cannot be excluded. When we applied further adjustment for BMI, marital status, income quartiles, self-reported health and previously diagnosed diabetes the associations remained similar to our main findings (Supplementary table S2). Nonetheless, potential confounders, such as physical activity, sedentary behaviour, as well as other food sources of saturated fat and dietary habits other than fat/oil intake may also influence these associations and need to be considered in future analyses.

On the other hand, this study also has important strengths. This is one of the few analyses that used household-level food availability data to examine the association between dietary habits and chronic disease outcomes. HBSs are frequently used to assess nutritional profiles of populations in nationally representative surveys, and they have several advantages, including affordability and international comparability.^26^ Replication of our analysis in other settings and with various food items is recommended. This would not only provide more robust evidence for the substantive findings but would also confirm the value of HBS data in the research of diet–disease relationships.

Our findings add to the growing evidence on the relationship between dietary habits and CVD risk in Eastern European populations, and suggests that animal fat intake have contributed to the increased CVD risk in this region. Considering the modifiable nature of unhealthy diet and the high disease burden that stems from it in any population,^27^ this area of research is particularly important in order to provide evidence for public health nutritional interventions in Eastern Europe and beyond.

## Supplementary data

Supplementary data are available at *EURPUB* online.

## Supplementary Material

ckab128_Supplementary_Data

## References

[1] Micha R, Khatibzadeh S, Shi P, et al Global, regional, and national consumption levels of dietary fats and oils in 1990 and 2010: a systematic analysis including 266 country-specific nutrition surveys. BMJ 2014;348:g2272. 24736206 10.1136/bmj.g2272 PMC3987052

[2] Szostak WB, Sekuła W. Nutritional implications of political and economic changes in Eastern Europe. Proc Nutr Soc 1991;50:687–93. 1809976 10.1079/pns19910082

[3] Lunze K, Yurasova E, Idrisov B, et al Food security and nutrition in the Russian Federation - a health policy analysis. Glob Health Action 2015;8:27537. 26112143 10.3402/gha.v8.27537 PMC4481043

[4] Stefler D, Brett D, Sarkadi-Nagy E, et al Traditional Eastern European diet and mortality: prospective evidence from the HAPIEE study. Eur J Nutr 2021;60:1091–100. 32613328 10.1007/s00394-020-02319-9 PMC7900332

[5] Zatonski WA, HEM project team. Epidemiological analysis of health situation development in Europe and its causes until 1990. Ann Agric Environ Med 2011;18:194–202. 22324071

[6] Food and Agriculture Organisation of the United Nations (2019) FAOSTAT. Available at: http://www.fao.org/faostat/en/#home (21 October 2020, date last accessed).

[7] Bobak M, Skodova Z, Pisa Z, et al Political changes and trends in cardiovascular risk factors in the Czech Republic, 1985-92. J Epidemiol Community Health 1997;51:272–7. 9229056 10.1136/jech.51.3.272 PMC1060472

[8] Zatonski WA, Willett W. Changes in dietary fat and declining coronary heart disease in Poland: population based study. BMJ 2005;331:187–8. 16037448 10.1136/bmj.331.7510.187 PMC1179759

[9] Becker W. Comparability of household and individual food consumption data–evidence from Sweden. Public Health Nutr 2001;4:1177–82. 11924944

[10] Sekula W, Nelson M, Figurska K, et al Comparison between household budget survey and 24-hour recall data in a nationally representative sample of Polish households. Public Health Nutr 2005;8:430–9. 15975190 10.1079/phn2004695

[11] de Oliveira DCRS, de Moura Souza A, Levy RB, et al Comparison between household food purchase and individual food consumption in Brazil. Public Health Nutr 2019;22:841–7. 30522532 10.1017/S1368980018002987 PMC10260444

[12] Kozyreva P, Kosolapov M, Popkin BM. Data resource profile: The Russia Longitudinal Monitoring Survey-Higher School of Economics (RLMS-HSE) Phase II: monitoring the economic and health situation in Russia, 1994-2013. Int J Epidemiol 2016;45:395–401. 26874929 10.1093/ije/dyv357 PMC5007614

[13] National Research University Higher School of Economics. Russian Longitudinal Monitoring Survey - HSE. Available at: https://www.hse.ru/en/rlms/ (2 December 2020, date last accessed).

[14] Carolina Population Center, University of North Carolina at Chapel Hill. The Russia Longitudinal Monitoring Survey – Higher School of Economics. Available at: https://www.cpc.unc.edu/projects/rlms-hse (2 December 2020, date last accessed).

[15] Rabe-Hesketh S, Skrondal A. Discrete-time survival. In: Multilevel and Longitudinal Modelling Using Stata, Vol II: Categorical Responses, Counts and Survival, 3rd edn. College Station, TX: Stata Press, 2012: 749–89.

[16] Schwingshackl L, Bogensberger B, Bencic A, et al Effects of oils and solid fats on blood lipids: a systematic review and network meta-analysis. J Lipid Res 2018;59:1771–82. 30006369 10.1194/jlr.P085522 PMC6121943

[17] Benhizia F, Hainault I, Serougne C, et al Effects of a fish oil-lard diet on rat plasma lipoproteins, liver FAS, and lipolytic enzymes. Am J Physiol 1994;267:E975–82. 7810643 10.1152/ajpendo.1994.267.6.E975

[18] Pimpin L, Wu JHY, Haskelberg H, et al Is butter back? a systematic review and meta-analysis of butter consumption and risk of cardiovascular disease, diabetes, and total mortality. PLoS One 2016;11:e0158118. 27355649 10.1371/journal.pone.0158118 PMC4927102

[19] Liu Q, Rossouw JE, Roberts MB, et al Theoretical effects of substituting butter with margarine on risk of cardiovascular disease. Epidemiology 2017;28:145–56. 27648593 10.1097/EDE.0000000000000557 PMC5480968

[20] Dehghan M, Mente A, Rangarajan S, et al Association of dairy intake with cardiovascular disease and mortality in 21 countries from five continents (PURE): a prospective cohort study. Lancet 2018;392:2288–97. 30217460 10.1016/S0140-6736(18)31812-9

[21] Pala V, Sieri S, Chiodini P, et al Associations of dairy product consumption with mortality in the European Prospective Investigation Into Cancer and Nutrition (EPIC)-Italy cohort. Am J Clin Nutr 2019;110:1220–30. 31435641 10.1093/ajcn/nqz183

[22] DuBroff R, de Lorgeril M. Fat or fiction: the diet-heart hypothesis. BMJ Evid Based Med 2019;26:3–7. 10.1136/bmjebm-2019-111180 31142556

[23] Dietary Guidelines Advisory Committee. Scientific Report of the 2015 Dietary Guidelines Advisory Committee: Advisory Report to the Secretary of Health and Human Services and the Secretary of Agriculture. Washington DC: Department of Agriculture, Agricultural Research Service, 2015.

[24] Biro G, Hulshof KFAM, Ovesen L, et al Selection of methodology to assess food intake. Eur J Clin Nutr 2002;56 Suppl 2:S25–32. 10.1038/sj.ejcn.1601426 12082515

[25] Okura Y, Urban LH, Mahoney DW, et al Agreement between self-report questionnaires and medical record data was substantial for diabetes, hypertension, myocardial infarction and stroke but not for heart failure. J Clin Epidemiol 2004;57:1096–103. 15528061 10.1016/j.jclinepi.2004.04.005

[26] Lagiou P, Trichopoulou A, DAFNE contributors. The DAFNE Initiative: the methodology for assessing dietary patterns across Europe using household budget survey data. Public Health Nutr 2001;4:1135–41. 11924937

[27] GBD 2017 Diet Collaborators. Health effects of dietary risks in 195 countries, 1990-2017: a systematic analysis for the Global Burden of Disease Study 2017. Lancet 2019;393:1958–72. 30954305 10.1016/S0140-6736(19)30041-8 PMC6899507

