# Supplementary material for: Household availability of dietary fats and cardiovascular disease and mortality: prospective evidence from Russia
Source: Eur J Public Health. 2021 Jul 30;31(5):1037–41. doi: 10.1093/eurpub/ckab128 (PMC8565488; doi:10.1093/eurpub/ckab128)
Supplement: ckab128_Supplementary_Data [file ckab128_supplementary_data.pdf]

Supplementary table S1. Association between lard, butter, margarine and vegetable oil availability and self-reported CVD or death after excluding participants who died or reported CVD in the first two years of follow-up (n=6402)

Outcome	Food product	Availability	n outcome per 1000 person-years	OR	(95%CI)
Combined CVD incidence and all-cause mortality	Lard	No	1423/71.9	1.00	(ref.)
		Low	69/3.5	1.09	(0.85-1.40)
		High	79/2.5	1.33	(1.07-1.65)
	Butter	No	959/48.0	1.00	(ref.)
		Low	302/16.6	1.07	(0.93-1.23)
		High	310/13.4	1.04	(0.91-1.19)
	Margarine	No	1382/68.0	1.00	(ref.)
		Low	113/6.5	1.06	(0.85-1.31)
		High	76/3.4	1.04	(0.85-1.26)
	Vegetable oil	No	1110/57.7	1.00	(ref.)
		Low	221/10.0	1.15	(0.99-1.33)
		High	240/10.2	0.96	(0.83-1.11)
CVD incidence	Lard	No	620/71.9	1.00	(ref.)
		Low	30/3.5	1.12	(0.78-1.61)
		High	39/2.5	1.51	(1.07-2.12)
	Butter	No	420/48.0	1.00	(ref.)
		Low	120/16.6	0.97	(0.79-1.20)
		High	149/13.4	1.09	(0.90-1.33)
	Margarine	No	608/68.0	1.00	(ref.)
		Low	46/6.5	0.91	(0.68-1.23)
		High	35/3.4	1.15	(0.81-1.64)
	Vegetable oil	No	484/57.7	1.00	(ref.)
		Low	87/10.0	1.04	(0.82-1.31)
		High	118/10.2	1.05	(0.85-1.29)
All-cause mortality	Lard	No	975/75.3	1.00	(ref.)
		Low	44/3.6	1.02	(0.76-1.37)
		High	48/2.7	1.14	(0.88-1.48)
	Butter	No	651/50.3	1.00	(ref.)
		Low	223/17.2	1.19	(1.02-1.40)
		High	193/14.2	0.98	(0.83-1.15)
	Margarine	No	939/71.3	1.00	(ref.)
		Low	81/6.7	1.07	(0.85-1.36)
		High	47/3.6	1.03	(0.80-1.33)
	Vegetable oil	No	754/60.3	1.00	(ref.)
		Low	162/10.5	1.24	(1.04-1.48)
		High	151/10.9	0.90	(0.76-1.07)

All ORs are adjusted for age, sex, education, smoking and alcohol intake frequency. The four dietary fats/oils were also adjusted for each other

Supplementary table S2 Association between lard, butter, margarine and vegetable oil availability and self-reported cardiovascular disease or death after adjustment for further baseline characteristics of participants (n=5833)

Food product	Availability	n CVD or death/1000 person-years	model 1		model 2		model 3		model 4	
			OR	(95%CI)	OR	(95%CI)	OR	(95%CI)	OR	(95%CI)
Lard	No	1426/64.4	1.00	(ref.)	1.00	(ref.)	1.00	(ref.)	1.00	(ref.)
	Low	52/2.5	1.05	(0.83-1.33)	1.08	(0.85-1.38)	1.07	(0.84-1.37)	1.10	(0.86-1.41)
	High	101/3.0	1.27	(1.01-1.60)	1.30	(1.03-1.64)	1.28	(1.02-1.61)	1.30	(1.03-1.65)
Butter	No	963/42.7	1.00	(ref.)	1.00	(ref.)	1.00	(ref.)	1.00	(ref.)
	Low	357/17.3	1.03	(0.90-1.18)	1.02	(0.89-1.17)	1.02	(0.89-1.17)	1.02	(0.89-1.18)
	High	259/9.9	1.02	(0.89-1.16)	1.03	(0.90-1.18)	1.02	(0.89-1.16)	1.02	(0.89-1.17)
Margarine	No	1378/60.7	1.00	(ref.)	1.00	(ref.)	1.00	(ref.)	1.00	(ref.)
	Low	173/7.4	1.02	(0.84-1.23)	1.03	(0.85-1.25)	1.04	(0.85-1.26)	1.04	(0.86-1.27)
	High	28/1.7	1.19	(0.93-1.51)	1.23	(0.96-1.57)	1.21	(0.94-1.55)	1.19	(0.92-1.53)
Vegetable oil	No	1110/51.5	1.00	(ref.)	1.00	(ref.)	1.00	(ref.)	1.00	(ref.)
	Low	163/6.4	1.14	(0.98-1.33)	1.10	(0.94-1.28)	1.11	(0.95-1.29)	1.12	(0.96-1.30)
	High	306/12.0	0.93	(0.80-1.08)	0.94	(0.81-1.09)	0.95	(0.82-1.10)	0.94	(0.81-1.09)

Model 1: adjusted for age and sex

Model 2: in addition to model 1, further adjusted for education, smoking, alcohol intake, and the four dietary fats/oils for each other

Model 3: in addition to model 2, further adjusted for BMI

Model 4: in addition to model 3, further adjusted marital status, income quartiles, self-reported health, self-reported diabetes and geographical site of data collection
